# Uterine Involvement in Klippel–Trenaunay Syndrome: A Rare But Relevant Event. Review of the Literature

**DOI:** 10.3389/fsurg.2022.893320

**Published:** 2022-05-12

**Authors:** Gaspare Cucinella, Giuseppe Di Buono, Girolamo Geraci, Federica Ricupati, Giuseppe Gullo, Elisa Maienza, Giorgio Romano, Giulia Bonventre, Giuseppe Amato, Giorgio Romano, Salvatore Buscemi, Antonino Agrusa

**Affiliations:** ^1^ Obstetrics and Gynecology, “Villa Sofia Cervello” Hospital, University of Palermo, Palermo, Italy; ^2^ Department of Surgical, Oncological and Oral Sciences, University of Palermo, Palermo, Italy

**Keywords:** Uterus, uterine bleeding, Klippel–Trenaunay syndrome, genitourinary, gynecological surgery

## Abstract

**Introduction:**

Klippel–Trenaunay syndrome (KTS) is a rare vascular congenital disorder characterized by the classical triad of port-wine stains, abnormal growth of soft tissues and bones, and vascular malformations. The involvement of the genitourinary tract and of the uterus in particular is extremely infrequent but relevant for possible consequences.

**Methods:**

We performed an extensive review of the literature using the Pubmed, Scopus and ISI web of knowledge database to identify all cases of KTS with uterine involvement. The search was done using the MeSH term “Klippel–Trenaunay syndrome” AND “uterine” OR “uterus.” We considered publications only in the English language with no limits of time. We selected a total of 11 records of KTS with uterine involvement, including those affecting pregnant women.

**Results:**

Klippel–Trenaunay syndrome was described for the first time in the year 1900 in two patients with hemangiomatous lesions of the skin associated with varicose veins and asymmetric soft tissue and bone hypertrophy. Uterine involvement is a rare condition and can cause severe menorrhagia. Diagnosis is based on physical signs and symptoms. CT scans and MRI are first-choice test procedures to evaluate both the extension of the lesion and the infiltration of deeper tissues before treatment. The management of Klippel–Trenaunay syndrome should be personalized using careful diagnosis, prevention and treatment of complications.

**Conclusion:**

Klippel–Trenaunay syndrome is a rare vascular malformation with a wide variability of manifestations. There are no univocal and clear guidelines that suggest the most adequate monitoring of the possible complications of the disease. Treatment is generally conservative, but in case of recurrent bleeding, surgery may be needed.

## Introduction

Klippel–Trenaunay syndrome (KTS) is a rare vascular congenital disorder. It is associated with the classical triad of port-wine stains, abnormal overgrowth of soft tissues and bones, and vascular malformations (1). KTS involves most frequently the lower limbs and less commonly the upper extremity and trunk (2). Visceral involvement is rare but has been described in the colon, small bowel, bladder, kidney, spleen, liver, mediastinum and brain. We can also see finger anomalies and visceromegaly. The involvement of the genitourinary tract is extremely rare. In this article, we present an extensive review of the literature on patients with KTS and genital involvement.

## Materials and Methods

**Figure 1:**
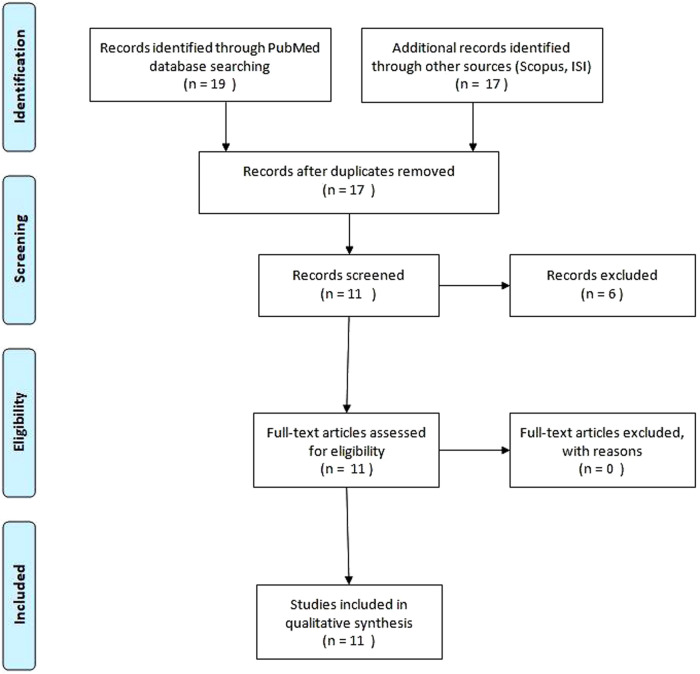
Systematic literature review to identify studies about Klippel–Trenaunay syndrome and uterine involvement.

We performed an extensive review of the literature using the Pubmed, Scopus and ISI web of knowledge database to identify all cases of KTS with uterine involvement. The search was done using the MeSH term “Klippel–Trenaunay syndrome” AND “uterine” OR “uterus.” We considered publications only in the English language with no limits of time. We selected a total of 11 records of KTS with uterine involvement, including those affecting pregnant women (Figure 1). The data on patients are reported in Table 1 and include age, limb or trunk involvement, the presence of internal lesions and the management of vascular lesions.

**Table 1:** Uterine involvement of Klippel–Trenaunay syndrome and respective treatments.

Study	Characteristics	Age	Limb involvement	Trunk involvement	Internal lesions	Treatment
Aronoff et al.	Female	30	Left upper extremities Left psoas muscle	Chest wall, arms	Spleen, uterus	Hysterectomy
Markos et al.	Female	26	Right lower limb knee, hip	–	Uterus	Hormonal with levonorgestrel
Milman et al.	Female	45	–	–	Uterus	Total hysterectomy and bilateral salpingectomy VLS
Rodriguez Peña et al.	Female	14	Lower left limb that extended to the pelvic region.	–	Bladder and uterus	Selective arterial embolization of the internal and external iliac territories//laser endo-coagulation
Zhang et al.	Female in pregnancy	24	Left buttock left lower extremity	–	Vulva Vagina	Laparotomy followed by embolization of the bilateral internal iliac artery
Yara et al.	Female in pregnancy	23	Left leg	–	Uterus	Elective cesarean section
Sun et al.	Female in post menopause	55	Left leg with arteriovenous shunts	–	Vaginal wall	Interventional arteriovenous fistula occlusion operation
Verheijen, et al.	Female in pregnancy	28	Right popliteal region	–	Uterus	Cesarean section
Lawlor et al.	Female	25	Right lower extremity anal region and perineal skin	–	Uterus	Hormonal with levonorgestrel
Watermayer et al. (3)	Female in pregnancy	21	Left lower limb	–	Vulva, cervix and uterus	Cesarean section
Minguez et al. (4)	Female	31	Lower limb	–	Vulva and uterus	Sclerotherapy

## Results

In this review of the literature, a total of 11 patients with KTS and uterine involvement were evaluated. Their bladder and uterus were the most commonly affected organs, and all of them had a positive history of intermittent bleeding that manifested in the form of hematuria or menometrorrhagia. In 10 cases, we registered uterine and vaginal involvement. All patients had a history of severe menometrorrhagia with significant vascular abnormalities of the reproductive organs. Only in the cases reported by Markos (5) and Lawlor and Charles-Holmes (6), hormonal therapy with levonorgestrel alone was effective. In the other cases, conservative management failed, and the patients were treated with surgical procedures. In particular, the failure of conservative treatment included recurrent bleeding requiring repetitive hospitalization or massive hemorrhage resistant to transfusion. Aronoff and Roshon (7) described the case of a 30-year-old woman with KTS and Kasabach–Merritt syndrome who had disseminated intravascular coagulation (DIC) with severe hemorrhage. The radiologic embolization of uterine arteries and subsequent abdominal hysterectomy was ineffective, and finally, bleeding was controlled with the use of intravenous low-dose heparin and antithrombin III. Milman et al. (8) described the case of a 45-year-old woman with KTS with abnormal uterine bleeding treated with total laparoscopic hysterectomy because of the smaller size of her uterus (15.7 × 6.9 × 5.3 cm). Rodriguez Peña and Ovando et al. (9) exposed the case of a 14-year-old female patient with macroscopic hematuria and metrorrhagia due to the presence of multiple angiomatous lesions in the bladder managed with a selective arterial embolization of the internal and external iliac territories and then with laser endo-coagulation of the bleeding foci. Metrorrhagia instead was controlled by the use of luteinizing hormone releasing hormone (LHRH) analogs. In nonpregnant women, postmenopausal vaginal bleeding from vaginal varices rarely occurs and is typically related to gynecological malignancies (10–12). Sun et al. (13) described a rare case of vaginal bleeding secondary to increased central venous pressure due to the presence of arteriovenous shunts in the leg of the patient. An arterial angiography was performed, and the arteriovenous shunts were partly closed by an interventional arteriovenous fistula occlusion operation. In the literature, a few cases of pregnancy in patients with KTS have been reported. Zhang et al. presented the case of a late puerperal hemorrhage in a patient with this syndrome, which became complicated with DIC, peritonitis and sepsis, and this patient was treated first with an emergency hysterectomy because of the occurrence of hemoperitoneum and later with bilateral internal iliac artery embolization to prevent the risk of recurrent bleeding (14). Yara et al. reported the case of a pregnant woman with KTS, complicated with diffuse venous malformation of the uterus, who underwent an elective cesarean section because of severe dystonia (15). Another case of pregnancy in a patient with KTS treated with an uncomplicated cesarean section was described by Verheijen et al. (16). This patient, in the course of her first pregnancy, developed a circumscript angiomatosis on the left and right sides of the uterus that extended into the cervix and that caused a slight growth retardation of the fetus. In KTS, the involvement of the urinary tract is up to 10% and hematuria, which occurs in more severe cases, is usually the initial clinical manifestation. Rubenwolf et al. (17) reported the clinical presentation and surgical management of a 9-year-old boy with extensive vascular malformations occupying more than two-thirds of the bladder lumen, which led to recurrent episodes of life-threatening gross hematuria. In this case, due to the extent of bladder involvement and the vulnerability of cystoscopy, conservative management was not possible, and surgical treatment with trigone sparing cystectomy and enterocystoplasty using an ileo-cecal segment was performed. Vulvar or vaginal vascular malformation is a rare manifestation of the KTS. Molkenboer and Hermans described the case of a woman with a vulvar swelling due to varicosis of the right labium removed by electrocoagulation (18).

## Discussion

KTS or angio-osteodystrophy syndrome has been listed as a “rare disease.” Its incidence is estimated between 2 and 5 in 100,000 and is found equally in both sexes (19). The age of onset of the first manifestations is birth or the first year of life. This condition was described for the first time in the year 1900 by two French Physicians, Maurice Klippel and Paul Trenaunay, in two patients with hemangiomatous lesions of the skin associated with varicose veins and asymmetric soft tissue and bone hypertrophy (20). Today, these three major findings constitute the primary diagnostic criteria of the syndrome. Later, Parkes-Weber added the presence of arteriovenous fistulas to the symptomatology of KTS, and it represented a distinct disorder called Parkes–Weber syndrome (21). The etiology of this syndrome is still unknown. Generally, the disease occurs sporadically with no sex predilection, although some authors have proposed that KTS affects males more often than females (2, 22, 23). Vahidnezhad et al. hypothesized that KTS may result from mutations in *PIK3CA*, the gene involved in the construction of the enzyme PI3K (phosphatidylinositol 3-kinase), cellular proliferation and migration, suggesting that KTS belonged to the PIK3CA-related overgrowth spectrum (PROS) (22, 24). Recently, several genetic studies reported sporadic translocations of chromosomes 5–11 and chromosomes 8–14 as the cause (25). Tian et al. proposed as a molecular pathogenic mechanism of the disease an angiogenic factor, called VG5Q, which causes an increase in angiogenesis when overexpressed (26). Patients with a mutation in the *PIK3CA* gene show an abnormal growth of the bones, soft tissues and blood vessels. The most common skin manifestation of the syndrome is represented by capillary malformations or “port-wine stains” that are often noted at birth. As reported by Jacob et al. in a study of 252 patients with KTS, capillary malformations (port-wine stains) were found in 246 patients (98%), varicosities or venous malformations in 182 (72%) and limb hypertrophy in 170 (67%), although the patients did not have all three symptoms at the same time (27). Capillary malformations are dilated telangiectatic vessels and appear in the upper dermis with a dermatomal distribution (28). These malformations do not tend to regress spontaneously with time but may cause hemorrhage and internal bleeding (29), especially in cases of visceral involvement such as the pleura, spleen, liver, bladder and colon (30, 31). Varicose veins are found in 76–100% of patients with KTS and may be distributed over extensive areas of the affected extremity, especially below the knee, on the lateral face of the thigh and occasionally in the pelvic region (31). Hypertrophy of the bones and soft tissues is present in a majority of patients with KTS and affects one or more limbs (32). Vascular abnormalities can also affect other organs, including the anterior chamber of the eye (glaucoma), retina (retinal varices, choroid angioma) and the central nervous system (the absence of deep cerebral veins, a dilation of the sinuses petrous and cavernous or hemimegalencephaly). Other central nervous system abnormalities are microcephaly, macrocephaly, cerebellar hemihypertrophy, cerebral atrophy and cerebral calcifications. Patients with the involvement of the gastrointestinal tract and the genitourinary tract have symptoms such as hematuria and/or hematochezia (33). The involvement of the gastrointestinal tract occurs in 20% of the patients and may be unnoticed in the absence of obvious signs. It most frequently affects the distal colon and the rectum, manifesting itself in the form of occult bleeding or massive hemorrhages (34). Genitourinary involvement occurs in severe cases, and vascular anomalies can affect external or visceral genitourinary systems. Furness et al. (35), in a study conducted on a large patient population with KTS, found that 23% with KTS (51 of 218) had cutaneous genitourinary involvement and 51% with KTS (111 of 218) had visceral genitourinary involvement with vascular malformations of the abdominal wall, pelvis or perineum. The presence of vascular abnormalities is not predictive of visceral involvement. For this reason, to avoid postoperative complications that may occur when these vascular abnormalities are inadvertently identified, all patients with KTS must be subjected to instrumental examinations for visceral vascular malformations before surgery. Gross hematuria from a visceral genitourinary source was reported in 1% of patients with KTS and is usually the first clinical sign of bladder involvement (34). Other sources of bleeding are external genitalia, urethra, kidney and ureter (35). The diagnosis is confirmed by radiological and cystoscopic studies. CT scan or MRI of the abdomen and pelvis evaluates the extent of vascular lesions and infiltration of deeper tissues. On cystoscopy, vascular lesions usually appear reddish blue, may appear sessile or pedunculated, flattened or lobulated and are more frequently localized on the anterior bladder wall and dome, but rarely in the trigone and the neck. During the procedure, a biopsy is not recommended because it can cause massive bleeding. The treatment of KTS-associated gross hematuria is still an object of study and is based on observation or surgical excision. Conservative management with intravenous hydration, transfusion and bladder irrigation using saline solution can be considered as the initial treatment for gross hematuria whenever possible. However, in the presence of refractory or life-threatening hematuria, a more invasive approach may be necessary. When vascular lesions are located extratrigonally, partial cystectomy is the standard surgical approach. Selective embolization of the internal iliac arteries can be a therapeutic alternative, but cases of relapse due to the development of collateral circulation and infarction of the bladder or prostate have been described in the literature (36). In refractory cases, laser coagulation and formalin instillation appear to be effective and should be attempted before any surgical intervention. Endoscopic management is not recommended as it may result in worsening hematuria (37). Uterine involvement is also an extremely rare condition, and there are only a few cases described in the literature. In a study of 218 patients with KTS conducted by Husmann et al. (33), only five patients had severe menorrhagia, and four of these had significant vaginal or uterine vascular abnormalities. Vascular lesions are usually confirmed by MRI, which evaluates the extension, the size and the anatomical relationships of the malformation. CT scan and/or MRI should always be performed before any therapeutic procedure, with the only exception of small and superficial localizations, for which ultrasonography is sufficient. Menorrhagia secondary to KTS vascular lesions can be well controlled with hormonal therapy (LHRH analogs and progestogens) (5), but in the presence of recurrent bleeding requiring repetitive hospitalization or massive hemorrhage resistant to transfusion, surgery may be needed. Hysterectomy is the treatment of choice in case of abnormal uterine bleeding. Laparoscopic hysterectomy represents a better surgical approach for less postoperative discomfort, a shorter hospital stay and a faster recovery, but in the presence of a very large uterus, open surgery remains the first-choice approach in most cases. Other factors that allow the feasibility of laparoscopy are the flexibility of the anterior abdominal wall, the residual intra-abdominal volume and the surgical experience of the team. Pregnancy is a rare event in women with this syndrome, and only a few cases are reported in the literature (14–16). It could become complicated with bleeding, DIC and thromboembolic events, so antithrombotic prophylaxis should be advised (38). In the event of complications arising during labor, an emergency cesarean section becomes necessary. How fertility can be preserved in order to avoid known vascular complications by surrogacy maternity has been reported. This is possible (3) using embryos obtained by a controlled ovarian stimulation and performing fertilization through intracytoplasmic sperm injection (ICSI). Usually to avoid ovarian hyperstimulation syndrome (OHSS), it is better to freeze all by the vitrification system using gonadotropin-releasing hormone antagonist administration the day before human chorionic gonadotropin trigger (4, 39–42).

## Conclusions

KTS is a very rare disease with a wide variability of manifestations. The typical vascular lesions are not progressive or proliferative, but the syndrome has a clinical course evolutionary type worsening in the course of life. The diagnosis is predominantly clinical based on careful anamnesis, physical signs and symptoms, but it must be integrated with radiographic examinations (such as the X-ray of the segment affected by vascular anomalies), with CT scan and MRI (which is the elective examination for vascular malformations), with a ultrasound of the internal organs. Although biopsy is contraindicated as it could cause massive bleeding, histology examination should be limited to cases where the imaging methods are not able to provide an accurate diagnosis and when a tumor lesion is suspected. The prognosis is linked to the presence of complications that appear to be more common when the cutaneous vascular anomalies are localized to the trunk or abdomen and have a map shape. In the literature, there are no univocal and clear guidelines that suggest the most adequate monitoring of the possible complications of the disease. Most of the authors focus on specific aspects of the syndrome such as urological and gynecological complications, on radiological diagnostics or on the genetic peculiarities of the disease. Management and diagnostic investigations of KTS should be personalized and guided by a multidisciplinary clinical evaluation of the patient. Treatment is generally conservative, but repeated transfusion and life-threatening bleeding episodes require surgical treatment. Certainly, a diversified and tailored multidisciplinary approach is necessary (43).
